# Chemical composition and microbiota changes across musk secretion stages of forest musk deer

**DOI:** 10.3389/fmicb.2024.1322316

**Published:** 2024-03-05

**Authors:** Zhongxian Xu, Feng Li, Qian Liu, Tianyuan Ma, Xiaolan Feng, Guijun Zhao, Dejun Zeng, Diyan Li, Hang Jie

**Affiliations:** ^1^Sichuan Wildlife Rehabilitation and Breeding Research Center, Key Laboratory of Southwest China Wildlife Resources Conservation (Ministry of Education), China West Normal University, Nanchong, China; ^2^Farm Animal Genetic Resources Exploration and Innovation Key Laboratory of Sichuan Province, Sichuan Agricultural University, Chengdu, China; ^3^Bio-resource Research and Utilization Joint Key Laboratory of Sichuan and Chongqing, Chongqing Institute of Medicinal Plant Cultivation, Chongqing College of Traditional Chinese Medicine, Chongqing, China

**Keywords:** forest musk deer, chemical composition, microbiota, musk secretion stage, mating state

## Abstract

Forest musk deer is the most important animal for natural musk production, and the musk composition changes periodically during musk secretion, accompanied by variation in the com-position of deer-symbiotic bacteria. GC-MS and 16S rRNA sequencing were conducted in this study, the dynamic changes to correlated chemical composition and the microbiota across musk secretion periods (prime musk secretion period, vigorous musk secretion period and late musk secretion period) were investigated by integrating its serum testosterone level in different mating states. Results showed that the testosterone level, musk composition and microbiota changed with annual cycle of musk secretion and affected by its mating state. Muscone and the testosterone level peaked at vigorous musk secretion period, and the microbiota of this stage was distinct from the other 2 periods. Actinobacteria, Firmicutes and Proteobacteria were dominant bacteria across musk secretion period. PICRUSt analysis demonstrated that bacteria were ubiquitous in musk pod and involved in the metabolism of antibiotics and terpenoids in musk. “Carbohydrates and amino acids,” “fatty acids and CoA” and “secretion of metabolites” were enriched at 3 periods, respectively. *Pseudomonas*, *Corynebacterium*, *Clostridium*, *Sulfuricurvum* were potential biomarkers across musk secretion. This study provides a more comprehensive understanding of genetic mechanism during musk secretion, emphasizing the importance of *Actinobacteria* and *Corynebacterium* in the synthesis of muscone and etiocholanone during musk secretion, which required further validation.

## 1 Introduction

Musk, a precious raw ingredient in traditional Chinese medicine and perfume manufacturing, is secreted from the glandular sac located between the navel and genitals of male forest musk deer (*Moschus berezovskii*) (FMD) (Meng et al., 2012). The sticky, white and malodorous liquid musk initially secreted by the musk gland is transported by catheters to a musk pod and matured to form blackish-brown solids with a specific musk odor by eventual fermentation (Bi et al., 1988). The initial liquid musk is synthesized during the prime musk secretion period (PMSP), a large amount of liquid musk is produced and forms semisolid musk during the vigorous musk secretion period (VMSP), and liquid musk stored in the musk pod undergoes full maturation and becomes the final solid musk during the late musk secretion period (LMSP) (Bai et al., 2013).

Testosterone (T) is the most essential factor targeting the testis to induce musk formation (Wilslocki et al., 1947; Wang et al., 2012; Bai et al., 2013; Fan et al., 2018). Studies showed that sex hormones (testosterone and estradiol) levels are consistent with annual cycle of musk formation and secretion (Zheng et al., 2019). Testosterone level increases at first and then decreases across the three stages of musk secretion (Bai et al., 2013; Suo et al., 2020), it reaches the peak at vigorous musk secretion period, and is significantly higher than other periods (Bai et al., 2013; Fan et al., 2018). Meanwhile, testosterone plays a major role in seasonal development of musk glands for musk-secreting animals like musk deer, muskrat, and Indian civet (Zhang et al., 2017). There is a positive correlation between musk yield and serum T levels, and negative correlation with serum estradiol and progesterone levels (Bai, 2012; Bai et al., 2013; Zhang et al., 2015). However, it remains unknown whether the T level is associated with mating status of musk deer in breeding season.

Jie has reported that the number of chemical compositions of musk ascended from June to August and declined from August to October, it varied with the physiological process of musk secretion. They also found ketones were dominant chemicals at vigorous musk secretion period in forest musk deer (Jie et al., 2021). Furthermore, our previous research showed that a greater amount of musk is produced in unmated males (UMs: 22.79 ± 3.6 g, *n* = 5) than mated males (MMs: 1.12 ± 0.34 g, *n* = 5), hinting that the production of musk is associated with its mating states (Li et al., 2016). Muscone, as a preponderant ingredient of natural musk, is claimed to function as pheromone for chemical communication for sexual attractant for females in rutting season (Sokolov et al., 1987), which was verified by more muscone are detected in UMs at LMSP (Li et al., 2016).

Studies have shown that core bacterial communities (Firmicutes, Bacteroidetes, and Proteobacteria) are shared by gut, fecal and musk microbiota in musk deer species, whereas the key genera for different species, mating states, ages, genders, and food-sources are distinct (Hu et al., 2017; Li et al., 2017; Hu et al., 2018; Li et al., 2018; Sun et al., 2019; Zhao et al., 2019). The fermentation hypothesis for mammalian chemical communication states that odorous metabolites secreted by fermentative bacteria in the scent glands can be used by the hosts for communication (Theis et al., 2013). Our study has proven that the maturation of musk is affected by interactions between the complex microbial community and chemical compounds produced by the host, and symbiotic bacteria underlie mating state-specific odors among animals is illustrated by the fact that many bacterial genera were overrepresented in UMs (Li et al., 2016).

## 2 Materials and methods

### 2.1 Animals and sample collection

A total of 30 healthy captive FMDs (15 UMs and 15 MMs) aged 2.5 to 6 years were raised in the Chongqing Institute of Medicinal Plant Cultivation (Chongqing, China). We collected the musk at the end of May to June, September, and October corresponding to PMSP, VMSP, LMSP. Six biological replicates for unmated and 7 biological replicates for mated individuals for each stage were sampled. All experiments were conducted in compliance with the guidelines approved by the Institutional Animal Care and Use Committee of Sichuan Agricultural University (approval ID: B20160403), and all efforts were made to minimize animal suffering. The blood samples were collected for serum T quantitative determination during musk secretion and non-secretion season (*n* = 10 for each group); body weight, musk yields were recorded at LMSP (*n* = 10). Serum testosterone was assayed by use of a testosterone ELISA kit (BNIBT, Beijing, China). The operation was conducted according to the specification.

### 2.2 Musk chemical component extraction and GC–MS determination

Sample preparation was performed as previously described with minor modifications (Li et al., 2016). Twenty milligrams of musk sample from each group were pooled, divided into 2 parts and dissolved in 2.5 mL of diethyl ether and ether alcohol, followed by a 2-h extraction by ultrasonication and centrifugation at 13,000 × *g* for 5 min. Two milliliters of supernatant was placed into a GC–MS instrument (GCMS-QP2010 Plus, Shanghai, China) for chemical composition detection with the chromatographic conditions as follows: column temperature: 40°C, inlet temperature: 290°C, interface temperature: 220°C, split injection with the pressure of 49.5 kPa. The column temperature program was as follows: 40°C (2 min), 200°C (5 min, 10°C/min), 240°C (5 min, 5°C/min), maintained at 290°C for 15 min. The total and column flow rates were 9.0 and 1.0 mL/min, respectively; the linear velocity was 36.1 cm/s; the purge flow rate was 3.0 mL/min; and the mass scanning range was 33–600 m/z. The acquired data in total ionic chromatograms (TICs) were compared with those in the mass spectral library of the National Institute of Standards and Technology (NIST). A confidence coefficient of above 80% was adopted for the data, and peak area % values were used to determine component variations. Finally, to discriminate the chemical compositions among 6 groups, we performed Partial Least Squares Discriminant Analysis (PLS-DA) after Pareto scaling.

### 2.3 Bacterial DNA isolation and 16S rRNA sequencing

Total bacterial DNA of each sample was isolated using an UltraClean Microbial DNA Isolation Kit (MOBIO, CA, USA) according to the manufacturer’s instructions. DNA quality was tested by 1.0% agarose gel electrophoresis and a NanoDrop spectrophotometer (NanoDrop Technologies, Wilmington, DE). The primers 515-F (5′- GTGCCAGCMGCCGCGG-3′) and 907-R (5’-CCGTCAATTCMTTTRAGTTT-3′) targeting the V3-V4 variable regions of the 16S-rRNA gene were used (Merrifield et al., 2013). The 16S-rRNA gene was amplified by PCR (95°C for 3 min, followed by 35 cycles of 95°C for 30 s, 55°C for 30 s, and 72°C for 45 s, and a final extension of 8 min at 72°C). The PCR products were purified and recovered by the Ezgene TM Gel/PCR Extraction Kit (Biomiga, USA) and submitted for library construction using the TruSeq Nano DNA LT Library Prep Kit (Illumina). Finally, a total of 39 libraries were constructed, and 250-bp paired-end (PE250) sequencing was performed on an Illumina Hiseq 2,500 platform (Illumina Inc., San Diego, CA, US) at Novogene (Beijing, China).

### 2.4 Bioinformatic analysis

The obtained low-quality raw reads and chimeras were removed using Cutadapt software (V1.9.1), the preprocessed paired-end reads were merged with FLASH (V1.2.7^1^) followed the criteria that the overlapped base of Read 1 and Read 2 was > = 10 bp and no mismatches allowed. High-quality reads were generated after quality control by FastQC (V0.11.7^2^). High-quality reads were assigned to operational taxonomic units (OTUs) with an unidentity threshold of 3% using UPARSE (V7.0.1001^4^) in QIIME (Quantitative Insights IntoMicrobial Ecology, Boulder, CO, USA, V1.8.0), and microbial taxa were identified from species annotation based on the GreenGenes reference database by Usearch software. We analyzed the relative abundances (RAs) of the top 10 phyla and top 20 genera, estimated four α diversity indices (Shannon, PD whole tree, observed OTU and Chao1), and compared the significant differences among 6 groups using ANOVA with Tukey’s HSD test. Prior to the evaluation of β diversity, unweighted pair-group method with arithmetic means (UPGMA) clustering trees were constructed to calculate Bray-Curtis and UniFrac distances, and canonical correspondence analysis (CCA) and principal coordinates analysis (PCoA) were implemented to evaluate the segregation of the microbial community using the R package (V2.15.3). Linear discriminant analysis (LDA) effect size (LEfSe) was performed to determine the differentially abundant microbes among the 6 groups (Segata et al., 2011), and selected bacteria with LDA score > 4 were used to calculate the Spearman correlation with the top 20 chemical components. Finally, the functional prediction of the musk microbiota was performed online using Phylogenetic Investigation of Communities by Reconstruction of Unobserved States (PICRUSt2) (Langille et al., 2013).

## 3 Results

### 3.1 The serum testosterone (T) level varies with musk secretion

**Figure 1:**
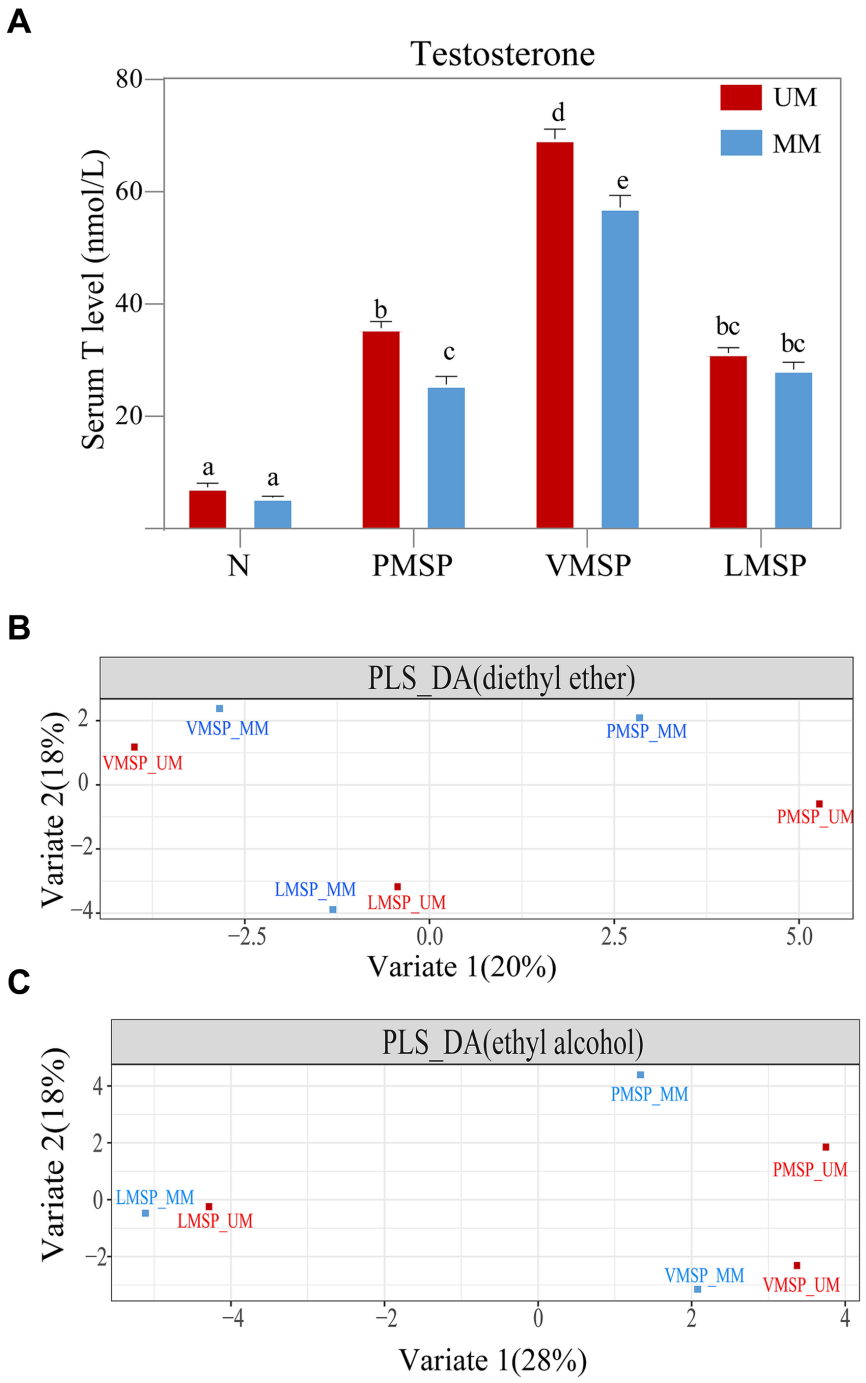
The serum testosterone and chemical composition features across musk secretion. **(A)** Histograms of serum testosterone levels and **(B,C)** partial least squares discriminant analysis (PLS-DA) across musk secretion based on diethyl ether and ethyl alcohol, respectively. **(A)** Different letters on the columns in panel A indicated significant differences, the same letters were nonsignificant, *p* < 0.05. N indicates non-musk secretion season. PMSP, VMSP, LMSP indicates prime, vigorous and late musk secretion period, respectively. UM and MM represents unmated and mated males, respectively.

The T concentration was significantly higher in the UM group (PMSP and VMSP) than that of MM group. The serum T concentration was at a basal level and as low as 7.37 ± 1.78 nmol/L and 5.54 ± 1.62 nmol/L in UMs and MMs, respectively, in non-secretion season (N). TheT level significantly increased from N to PMSP (35.72 ± 1.28 nmol/L in UMs and 25.68 ± 2.64 nmol/L in MMs), and reached a peak in VMSP (69.44 ± 3.24 nmol/L in UM and 57.24 ± 6.65 nmol/L in MM). It decreased gradually from VMSP to LMSP (31.32 ± 2.03 nmol/L in UMs and 28.34 ± 3.59 nmol/L in MMs) (Figure 1A). We collected 21.161 ± 5.434 g of fully mature secretions from UMs in the late musk secretion period, which was significantly greater than the amount derived from MMs (7.122 ± 1.648 g, *p* = 0.018). And the musk yields were significantly positive correlated (*R* = 0.719, *p* = 0.019) with serum T levels for MM, while no correlation was found for UM (Supplementary Figure S1).

### 3.2 The chemical composition changes across musk secretion

Generally, the TICs indicated that the number of chemicals extracted by diethyl ether (75.17 ± 8.32) was greater than that extracted by ethyl alcohol (66.00 ± 11.09); as the key medical active and aromatic component of natural musk, 3-methylcyclopentadecanone (muscone) was the most abundant component (26.99% in UM and 25.54% in MM), followed by 3a-hydroxy-5b-androstan-17-one (etiocholanone) (12.95% in UM and 12.30% in MM) and cholesterol (10.99 in UM and 7.82 in MM) (Supplementary Figures S2, S3; Supplementary Table S1). The PLS-DA results showed metabolites extracted by two solvents of LMSP were more distinct with PMSP and VMSP, and the differences between UM and MM were least within LMSP (Figures 1B,C).

### 3.3 Statistics of 16S rRNA sequencing data

In addition, we performed 16S rRNA sequencing to evaluate the bacterial dynamics, and a total of 1,869,786 high-quality reads of 39 samples were generated; an average of 52,868, 40,226 and 50,735 reads were obtained in PMSP, VMSP and LMSP; 45,202 and 51,141 high-quality reads were assigned to UMs and MMs, respectively (Supplementary Table S2). There were 1,431 OTUs that were assigned at the 97% similarity level; the average number of OTUs was 977 ± 64 in each group, ranging from 865 (PMSP-MM) to 1,014 (LMSP-UM), and there were 1,075 OTUs in PMSP, 1,157 OTUs in VMSP, and 1,248 OTUs in LMSP. The number of OTUs detected in UMs was significantly higher than that in MMs (~1,024 in UMs and ~ 930 in MMs, *p* = 0.0002). Taxonomic classification of the OTUs resulted in 39 phyla, 84 classes, 162 orders, 291 families, 569 genera and 654 species, among which, 391 out of 1,431 OTUs, 19 out of 39 phyla and 257 out of 569 genera were shared by all individuals within each group, and most unique OTUs were found in LMSP (37 in UMs and 14 in MMs) and VMSP (23 in MMs and 4 in UMs) (Supplementary Figure S4).

### 3.4 Different dominant microbiota composition at genus level

**Figure 2:**
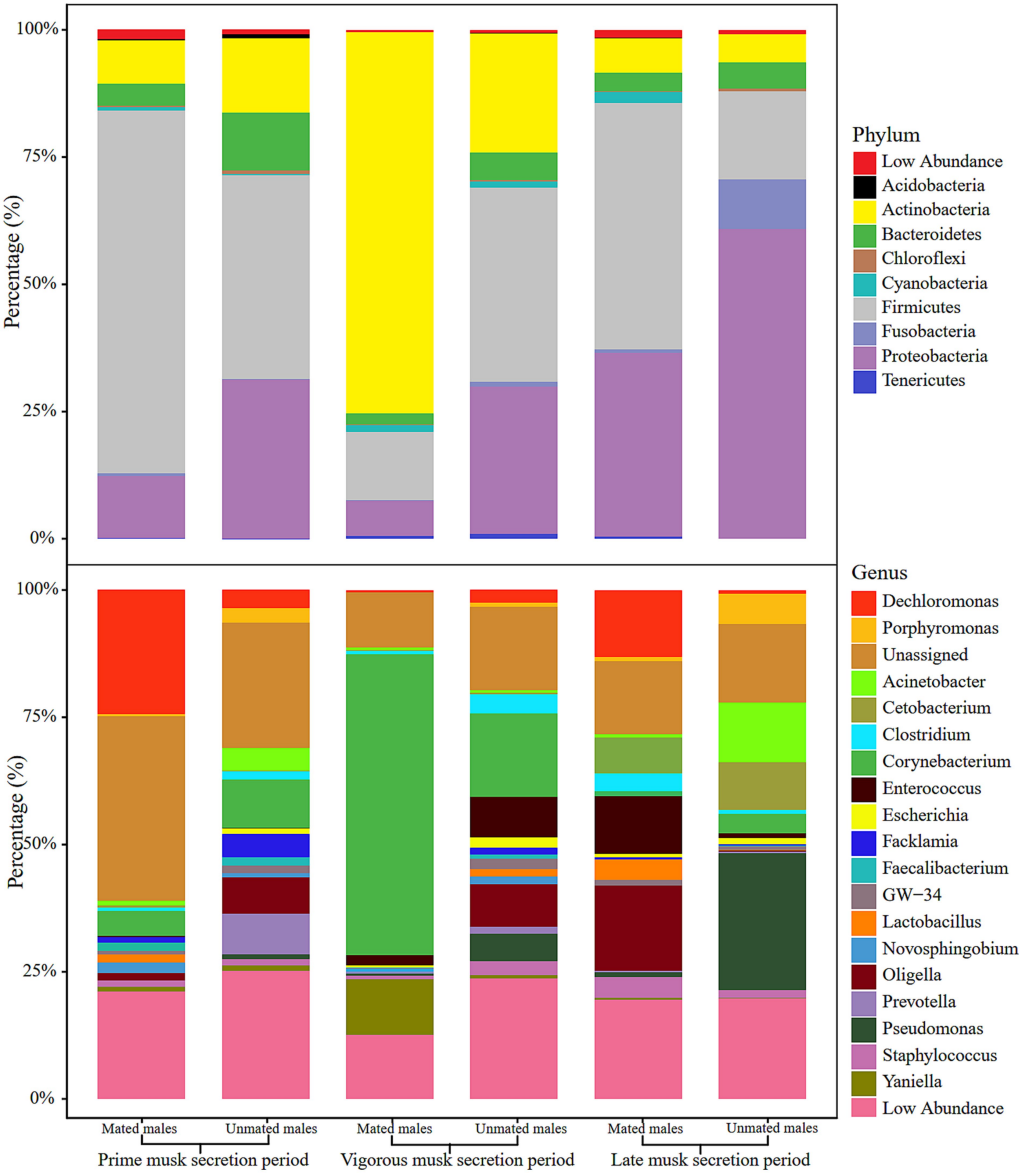
Histogram of relative abundance of microbiota composition at phylum (upper) and genus (lower) levels in the 6 groups.

We selected the top 10 phyla and top 20 genera in terms of relative proportions to illustrate the dominant microbiota composition. Actinobacteria, Firmicutes, Proteobacteria, and Bacteroidetes were common dominant microbial communities in the 6 groups, and their relative proportions accounted for more than 87% of all phyla (Figure 2 upper). Intriguingly, the relative proportion of Actinobacteria was as high as 77.57% ± 11.24% in the VMSP-MM group, which was much higher than that in UMs (20.39% ± 9.24%) and significantly different from that in the other 2 periods (*p* = 0.0017), indicating the absolute abundant role of Actinobacteria in this group. At the genus level, we found that *Corynebacterium* and an unassigned genus of Aerococcaceae were common dominant microbial communities during PMSP and VMSP. In particular, the genus *Corynebacterium* was an abundant dominant microbe (63.63%) in the VMSP-MM group, with a significantly higher (*p* = 0.0261) relative proportion than the average relative proportions in other groups (6.82%), as well as the relative proportion in UMs (14.21%). During LMSP, the main dominant genera were *Oligella*, *Enterococcus*, and *Cetobacterium* in MMs and *Pseudomonas*, *Cetobacterium*, and *Acinetobacter* in UMs (Figure 2 lower).

### 3.5 Microbiota diversity variations across musk secretion

**Figure 3:**
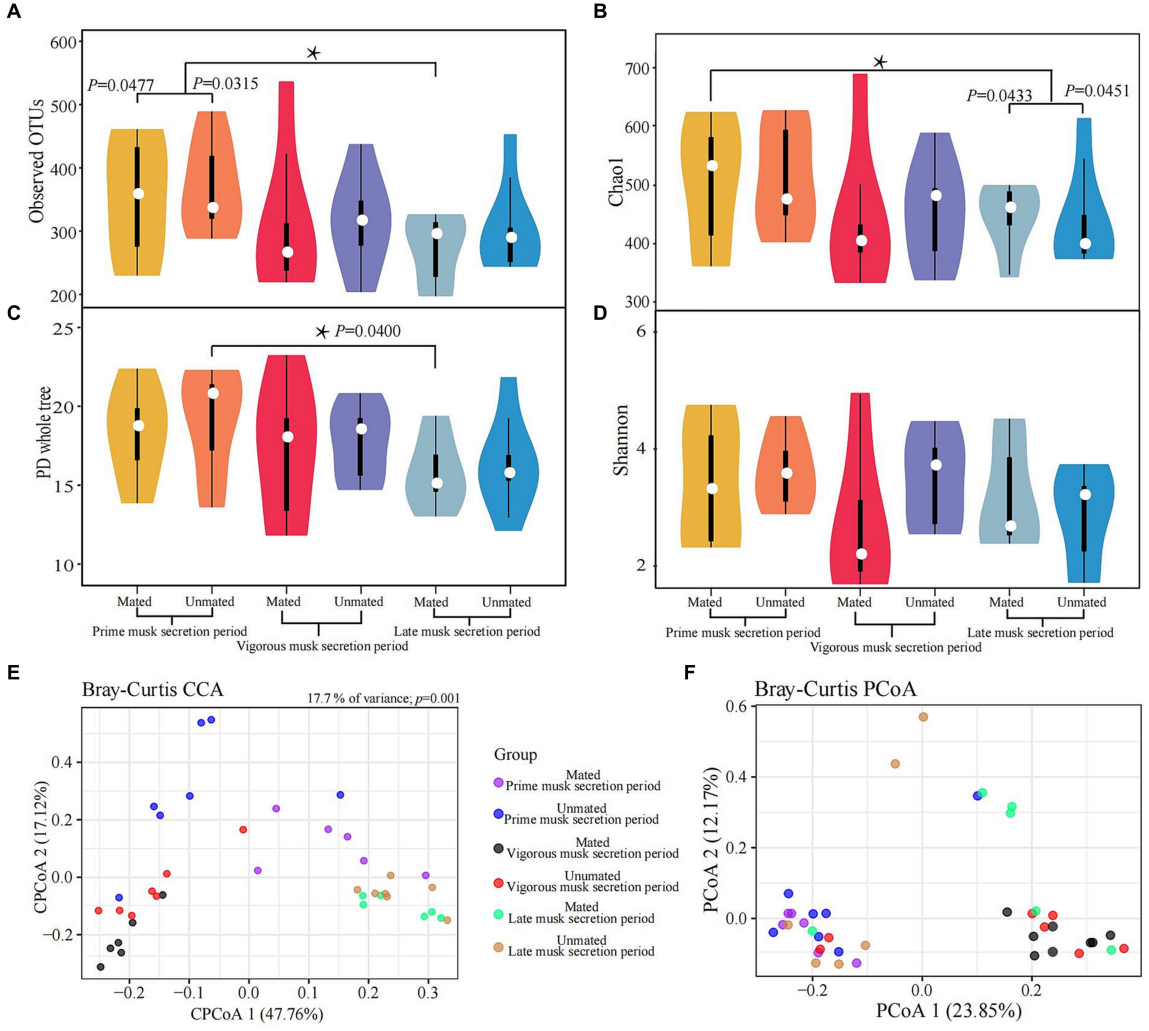
Variations in alpha **(A-D)** and beta **(E,F)** diversity of the musk microbiota. Asterisk means *p* < 0.05.

We calculated the α and β-diversity to further evaluate the differences in microbiota composition within and among the 6 groups. Overall, the α diversity showed negligible differences because of the inconsistent trends in variation across multiple metrices: the observed significant differences between PMSP and LMSP in MMs (*p* = 0.0477) for the observed OTU and Chao1 index (*p* = 0.0451) did not showed in PD whole tree and Shannon index (Figures 3A-D, Supplementary Table S3). The microbiota composition differences between groups based on Bray-Curtis distance are listed in Supplementary Table S3. A significant discrepancy between PMSP and VMSP was observed (*p* = 0.0040 in MM and 0.0100 in UM), variance between PMSP and LMSP was detected merely within MM (*p* = 0.0090). The CCA result explained 17.7% (*p* = 0.001) of the total variance between samples. All samples in VMSP were clustered and distinctly separated from the communities clustered by the majority of samples in PMSP and LMSP, which could be well explained by PCoA 1 (47.76%). However, this plot revealed negligible differences between the UM and MM groups. As expected, identical results for the musk microbiota in VMSP were revealed by the PCoA plot (Figures 3E,F).

### 3.6 Distinct bacteria interact with chemical components to mediate musk secretion

**Figure 4:**
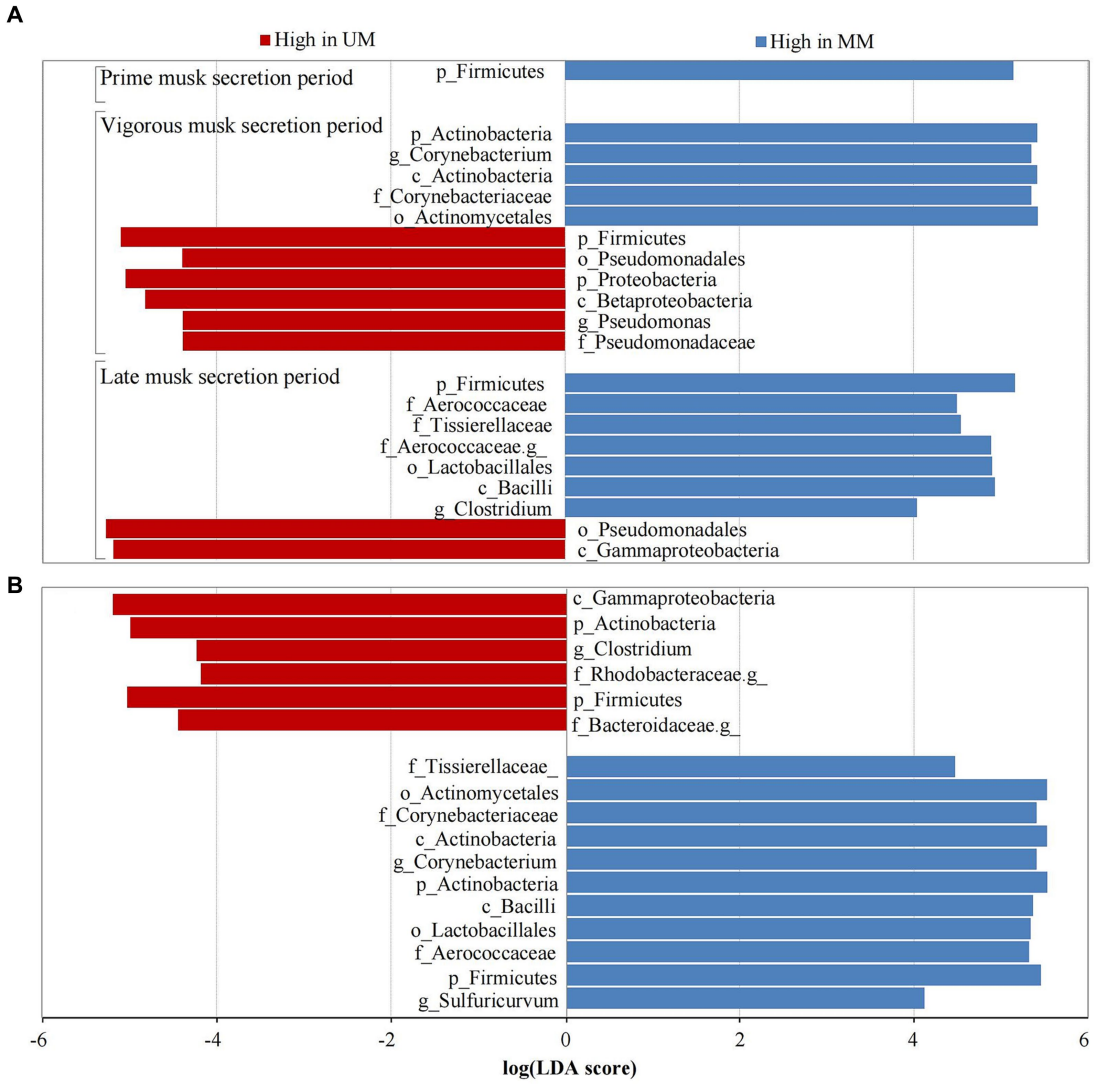
Taxa with significant difference (LDA > 4) across musk secretion stages **(A)** between unmated and mated males **(B)**. UM and MM represents unmated and mated males, respectively. A means LEfSe analysis of 21 significantly abundant taxa between Ums and MMs across 3 musk secretion stages. B means LEfSe analysis of 17 taxa were used as significant biomarkers between UMs and MMs.

We performed LEfSe analysis of the 6 groups to further determine differentially abundant bacterial taxa that might be considered biomarkers. Twenty-one taxa were identified as significantly abundant communities (LDA > 4 and *p* < 0.05) across 3 musk secretion stages, belonging to the top 3 dominant microbial communities (Firmicutes, Proteobacteria, and Actinobacteria). Among them, Firmicutes was dominant in MMs in the 3 periods (LDA > 5). During VMSP, 5 taxa of Proteobacteria including Proteobacteria, Betaproteobacteria, Pseudomonadales, Pseudomonadaceae, and *Pseudomonas*, and 5 taxa of Actinobacteria, including *Corynebacterium*, were distinct bacteria in UM and MM respectively; 6 taxa of Firmicutes (*Clostridium* and an unknown genus of Aerococcaceae) and 2 taxa of Proteobacteria were discrepant microbes in MMs and UMs during LMSP (Figure 4A). Furthermore, we identified 17 taxa as significant biomarkers (LDA > 4 and *p* < 0.05) between UMs and MMs, and more taxa were found in MMs, namely, 5 Firmicutes, 5 Actinobacteria and 1 Proteobacteria, particularly *Corynebacterium* and *Sulfuricurvum*. We identified 2 Firmicutes (including *Clostridium*), 1 Actinobacteria, 2 Proteobacteria (an unidentified genus of Rhodobacteraceae) and 1 Bacteroidetes (an unassigned genus of Bacteroidaceae) as biomarkers in UMs (Figure 4B).

**Figure 5:**
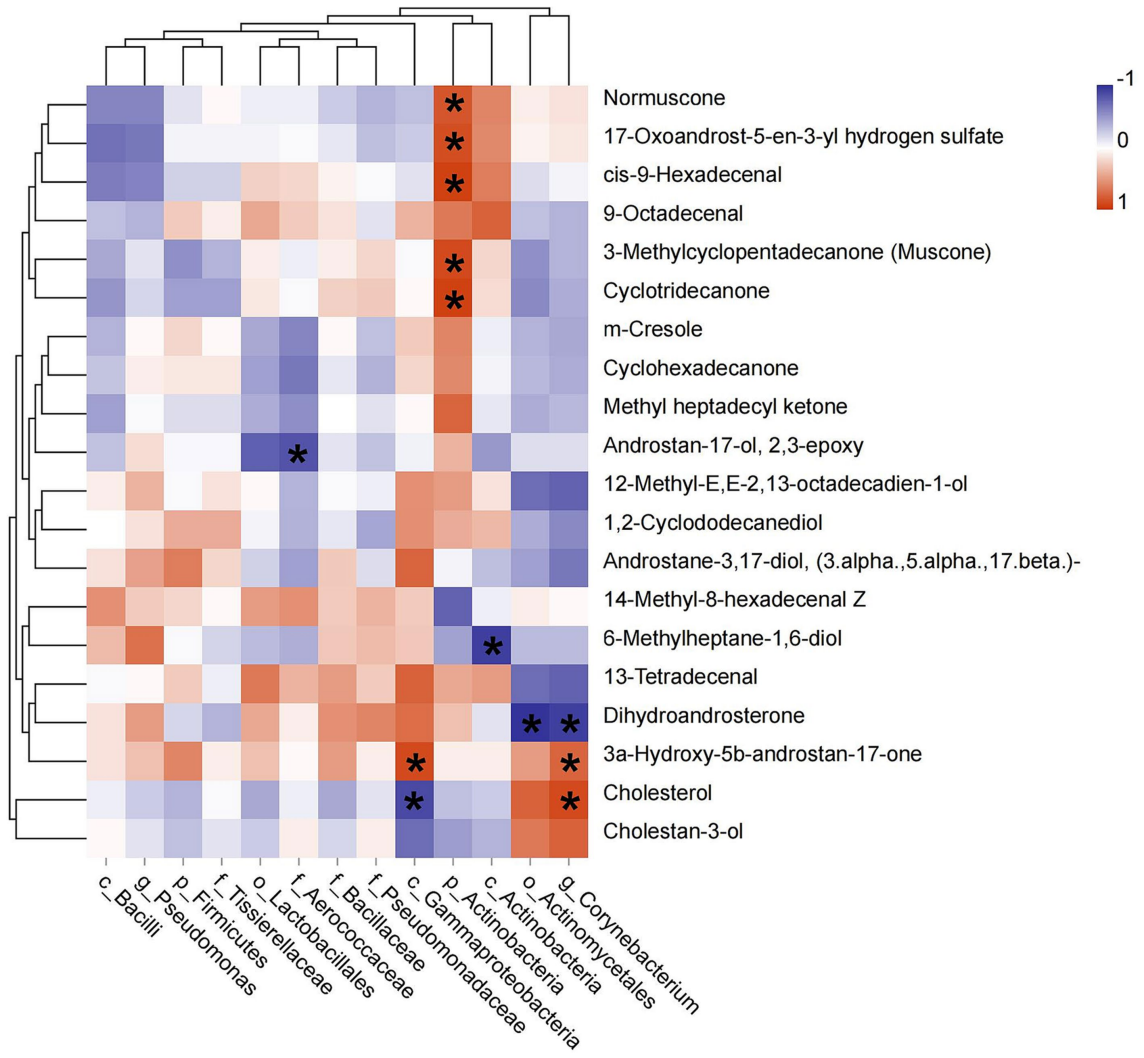
Pearson correlation between dominant bacterial taxa and the top 20 chemical components of musk. Asterisk means *p* < 0.05, red square indicates positive correlation and blue square indicates negative correlation.

The relative abundances of identified biomarkers were used to conduct spearman correlation analysis with the level of top 20 chemical components, we found that the abundance of p_Actinobacteria was positively correlated with muscone (*R* = 0.886, *p* = 0.019), normuscone (*R* = 0.829, *p* = 0.042), 17-Oxoandrost-5-en-3-yl hydrogen sulfate (*R* = 0.870, *p* = 0.024), cis-9-Hexadecenal (*R* = 0.928, *p* = 0.008) and cyclotridecanone (*R* = 0.928, *p* = 0.008). The abundance of c_Gammaproteobacteria had a positive relationship with 3a-Hydroxy-5b-androstan-17-one (etiocholanone) (*R* = −0.943, *p* = 0.005) and negative with cholesterol (*R* = −0.890, *p* = 0.019). The abundance of g_*Corynebacterium* was positively correlated with etiocholanone (*R* = 0.829, *p* = 0.042) and cholesterol (*R* = 0.886, *p* = 0.019), but negatively with dihydroandrosterone (*R* = −0.943, *p* = 0.005). And the abundance of f_Aerococcaceae was negatively correlated with androstan-17-ol, 2,3-epoxy (*R* = −0.845, *p* = 0.034) (Figure 5).

### 3.7 Function predictions of musk microbiota

**Figure 6:**
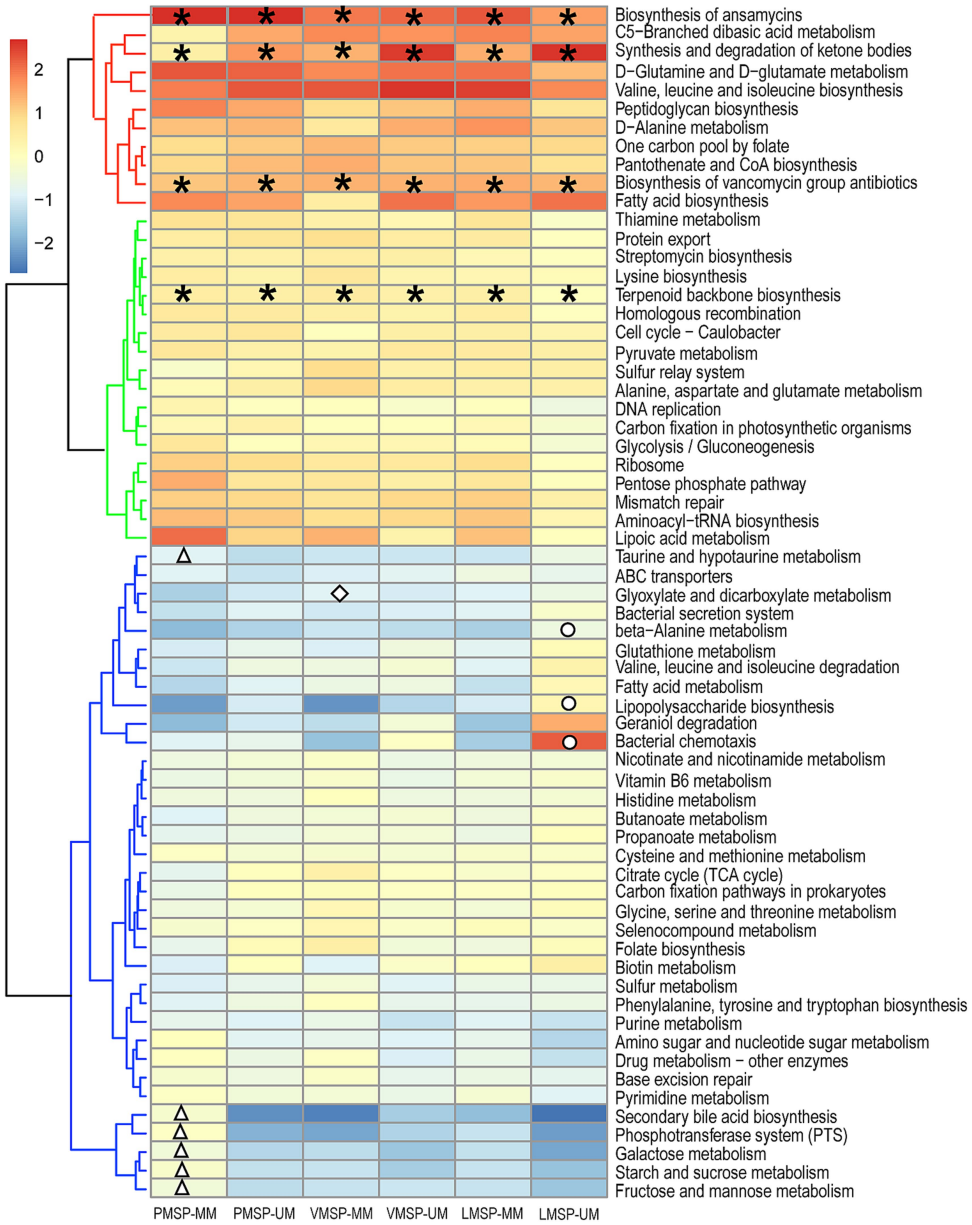
Heat map of the functional predictions of musk microbiota (normalized by Z-score). PMSP, VMSP, LMSP indicates prime, vigorous and late musk secretion period, respectively. UM and MM represents unmated and mated males, respectively. *represents pathways enriched in 6 groups, △ means PMSP-MM specific pathways, ◇ and ○ indicates VMSP-MM and LMSP-MM uniquely enriched pathways, respectively.

To explore the differences in microbial functions in each group, we used the assigned OTUs to determine the relative abundances of functional categories through PICRUSt2. We found 39 pathways shared predicted functions in the 6 groups. Intriguingly, “Biosynthesis of ansamycins (ko01051),” “Biosynthesis of vancomycin group antibiotics (ko01055)” and “Terpenoid backbone biosynthesis (ko00900)” belonging to metabolism of terpenoids and polyketides, as was “Synthesis and degradation of ketone bodies (ko00072)” were detected in all samples. For stage-specific functions, we found 6 pathways were specifically enriched in PMSP-MM. “Glyoxylate and dicarboxylate metabolism (ko00630)” was uniquely enriched in VMSP-MM. Three pathways were uniquely enriched in LMSP-UM. Moreover, “Geraniol degradation (ko00281)” was enriched in UMs during LMSP, and “Bacterial chemotaxis (ko02030)” was assigned only in the UM group across the 3 periods (Figure 6 and Supplementary Table S4).

## 4 Discussion

The hardship of sample collection and non-invasive principles of sampling limited the sample size in our study. The timid feature and furious reaction of forest musk deer increases the difficulty of sampling. Actually, the collection of musk at the initial stage would decrease the subsequent musk secretion, which would 39 sampling in the following stages. Therefore, it is not possible to collected enough musk of all individuals at one stage. Although we have studied the chemical composition and microbiota in UMs and MMs in our previous study, we merely elucidated the microbiota changes and the important roles of the dominant bacteria during LMSP (Li et al., 2018). Whether the T levels and dynamic composition of musk change with the bacterial communities colonizing the musk glands is unclear, and the relationship between mating states and microbiota across musk secretion stages need additional studies. We selected 30 healthy musk deer, sampled 6 unmated and 7 mated individuals during musk secretion. A total of 39 (18 UM and 21 MM) libraries were constructed for 16S rRNA sequencing. Moreover, due to the musk is one of the most valuable scented animal products and more expensive than gold. Sufficient musk (at least 0.8 g) for DNA extraction was priority, we pooled 20 mg musk of each group for GC–MS determination.

We further investigated the dynamic changes of chemical composition and the microbiota across musk secretion by integrating its serum T level in different mating states. As one of the most important androgens mediated by HPG axis, testosterone can affect the development and physiological responses of the musk gland (Bi et al., 1980; Knol, 1991). Bai found an obvious tendency of the fecal T levels in the musk-secreting period (2–7 years old’s male forest musk deer, *n* = 30), a similar pattern was found in estradiol but no obvious trend was detected in progesterone. Compared with the non-secretion season (114.15 ± 15.46 ng/g), T levels of PMSP (1731.50 ± 95.66 ng/g) and VMSP (3766.82 ± 98.48 ng/g) were higher significantly, which raised gradually before musk-secreting period (412.05 ± 54.14 ng/g), increased rapidly in PMSP, and peaked in VMSP, subsequently declined steeply in LMSP (616.79 ± 68.79 ng/g) (Bai, 2012; Bai et al., 2013). Results in this study showed that the serum T levels changed dynamically during musk secretion and peaked at VMSP as well, it increased significantly in musk secretion season and was much higher in UMs than MMs. Zhang et al. (2015) and Suo et al. (2020) reported the serum and fecal T levels increased first and then decreased in the three stages of secretion, respectively. In addition, we found a stronger positive correlation (*R*^2^ = 0.719, *p* = 0.019) in MM, but an uncorrelated relationship (*R*^2^ = 0.084, *p* = 0.816) in UM. Bai (2012) and Bai et al. (2013) indicated that musk yield was positively correlated with T level (*R*^2^ = 0.650, *p* < 0.05). Sun et al. (2020) discovered the musk secretion with higher T level was significantly more than that of with lower T level. Previous studies prompted the musk secretion through intramuscular injection of testosterone (Jie et al., 2014). Fan hinted that sex hormones might determine musk composition in early musk secretion, even facilitate the musk secretion (Fan et al., 2018). The muscone content showed the highest relative abundance among all components. Consistent with previous report (Su et al., 2009), steroids (cholesterol, cholestanol. and etiocholanone) presented at relatively high levels in musk.

Although there were some individual differences in alpha diversity there were no consistent trends in variation across multiple alpha metrics. The principal coordinate analysis of β diversity values illustrated samples in VMSP were distinctly separated from the other 2 periods, which were consistent with the highest T level observed at VMSP. It was speculated that the diversity of microbiota is affected by homeostasis in musk sacs maintained by hosts at different periods, and a slightly richer microbiota is essential for the synthesis of specific odor substances to make fully mature musk in the unmated males for more attractive to females (Li et al., 2016; Jie et al., 2021).

It is generally believed that substantial changes of the bacterial communities colonizing the musk gland are resulted from physiological activities during musk secretion (Li et al., 2018; Jie et al., 2019). Though Actinobacteria, Firmicutes, and Proteobacteria are dominant bacteria in musk deer (Sundset et al., 2007; Gruninger et al., 2014; Ishaq and Wright, 2014), their composition is dynamic across musk secretion, namely, Actinobacteria (especially there were 15 mated and 15 non mated deer in two groups) dominated in VMSP absolutely; Firmicutes and Proteobacteria accounted for majority of bacterial communities during PMSP and LMSP, respectively. Hu et al. have revealed the decline of Firmicutes-Bacteroidetes ratio with the seasonal variation but the dominant genera showed no significant changes of gut microbiota in forest musk deer (Hu et al., 2018). We have reported differences of relative microbiota abundance during forest musk deer development, Clostridiales and Bacteroidales were higher in juvenile, *Pseudomonas* and Lachnospiraceae were more abundant in the adult (Zhao et al., 2019). Li et al. (2021) have found the fecal microbiota composition in young forest musk deer at genus level changed from that found in 7–10 day’s old became stabilized after 30 day’s old, the relative abundance of *Actinobacteria*, *Spirochaetes*, *Ruminococcaceae_UCG-005*, *Treponema* and *Prevotella* was higher in the post-weaning than in the pre-weaning group (Li et al., 2020).

The LEfSe analysis showed that Firmicutes, which contain cellulolytic bacteria was the dominant bacteria across 3 stages (Hugenholtz, 2002). Proteobacteria which are resistant to antimicrobial compounds (Lee et al., 2012) and Actinobacteria which are the main sources of anti-inflammatory components (Scharf and Brakhage, 2013; O'Connor, 2015; Motoyama and Osada, 2016) in musk were dominant in VMSP. *Pseudomonas aeruginosa* can inhibit the growth of *Trueperella pyogenes* in FMD with abscess diseases (Yuan et al., 2020). Actinobacteria include members with significant economic and medical importance. Filamentous actinomycetes, such as *Corynebacterium* and *Streptomyces* species, can produce a plethora of bioactive secondary metabolites, many of these metabolic compounds are antimicrobial, anticancer, antiviral or immunosuppressive in activity (Prudence et al., 2020; Undabarrena et al., 2021; Seshadri et al., 2022). *Corynebacterium* is used as a commercial bacterial strain to synthesize heterologous terpenoids and as a model system for aromatic hydrocarbon metabolism research (Shen et al., 2012; Lee et al., 2016). The metabolites of *Corynebacterium* play analgesic, anticancer and antioxidant functions in therapy (Heider et al., 2012; Hari et al., 2019). These bacteria were confirmed to have positive correlations with muscone and etiocholanone (Gammaproteobacteria/*Corynebacterium* vs. etiocholanone and Actinobacteria vs. muscone). During LMSP, metabolites of Aerococcaceae might be the source of fatty acids and might protect against foreign bacterial invasion (Hugenholtz, 2002).

Pathways enriched in all groups illustrated that these bacteria are involved in the metabolism antibiotics and terpenoids, which are essential for the synthesis of antibacterial and anti-inflammatory components and specific odor substances in musk. For example, ansamycin and vancomycin are antibiotics used for the treatment of bacterial infections (Yu et al., 2002; Watanabe et al., 2003; Okano et al., 2017); terpenoids are the precursor of sterols and ketones (Mookherjee and Wilson, 1982; Wriessnegger and Pichler, 2013), and they are microbial metabolites of Streptomycetaceae (Hwang et al., 2014), Actinobacteria (*Actinomycetes* and *Corynebacterium*) (van Bergeijk et al., 2020), Myxobacteria (Schaberle et al., 2014) and Proteobacteria (*Escherichia coli*) in industry (Yamada et al., 2015; Lee et al., 2016; Wang et al., 2018). Among PMSP-specific pathways, the PTS is a major mechanism used by bacteria for the uptake of carbohydrates (Deutscher et al., 2008), which is related to the accumulation of carbohydrates and other amino acids at PMSP. Glyoxylate and dicarboxylate metabolism is associated with the biosynthesis of carbohydrates from fatty acids or CoA (Yoo et al., 2019). Bacterial secretion system has been shown to mediate protein export through the membranes of Gram-negative bacteria (King et al., 2009). Bacterial chemotaxis is an adaptive response to the environment, suggesting that UMs might be more attractive for females than MMs, and this attraction is driven by aromatic substances produced by bacteria in UMs (Miller et al., 2009).

## 5 Conclusion

To sum up, the serum T level, chemical composition and microbiota of musk exhibited dynamic changes with musk secretion. The microbiota of VMSP was distinct from the other 2 periods. Actinobacteria, Firmicutes and Proteobacteria are dominant bacteria across musk secretion, *Pseudomonas* and *Corynebacterium* were candidate biomarkers of the VMSP, *Clostridium* was a biomarker of the LMSP; *Corynebacterium* and *Sulfuricurvum* in MMs and *Clostridium* in UMs could be used to distinguish the mating state of FMD. We are also assuming that Actinobacteria and *Corynebacterium* have vital roles in the synthesis of muscone and etiocholanone during musk secretion, and further study is needed to confirm these hypotheses. However, these results predicted the metagenomic function, further validations were required to confirm our hypotheses.

## Data availability statement

The 16S rRNA sequencing data presented in the study are deposited at https://ngdc.cncb.ac.cn/bioproject, accession number PRJCA002782.

## Ethics statement

The animal study was approved by the Institutional Animal Care and Use Committee of Sichuan Agricultural University (approval ID: B20160403). The study was conducted in accordance with the local legislation and institutional requirements.

## Author contributions

ZX: Conceptualization, Methodology, Project administration, Writing – original draft, Writing – review & editing, Funding acquisition. FL: Supervision, Writing – review & editing, Software. QL: Writing – review & editing, Data curation, Formal analysis. TM: Data curation, Writing – review & editing, Visualization. XF: Writing – review & editing, Data curation, Investigation. GZ: Data curation, Writing – review & editing, Resources. DZ: Resources, Writing – review & editing, Software. DL: Software, Writing – review & editing, Data curation, Formal analysis. HJ: Writing – review & editing, Conceptualization, Funding acquisition, Project administration, Supervision.

## Footnotes

^1^ http://ccb.jhu.edu/software/FLASH/

^2^ https://www.bioinformatics.babraham.ac.uk/projects/fastqc/

^4^ http://drive5.com/uparse/
