# Supplementary figures and images for: Chemical composition and microbiota changes across musk secretion stages of forest musk deer
Source: Front Microbiol. 2024 Mar 5;15:1322316. doi: 10.3389/fmicb.2024.1322316 (PMC10948612; doi:10.3389/fmicb.2024.1322316)

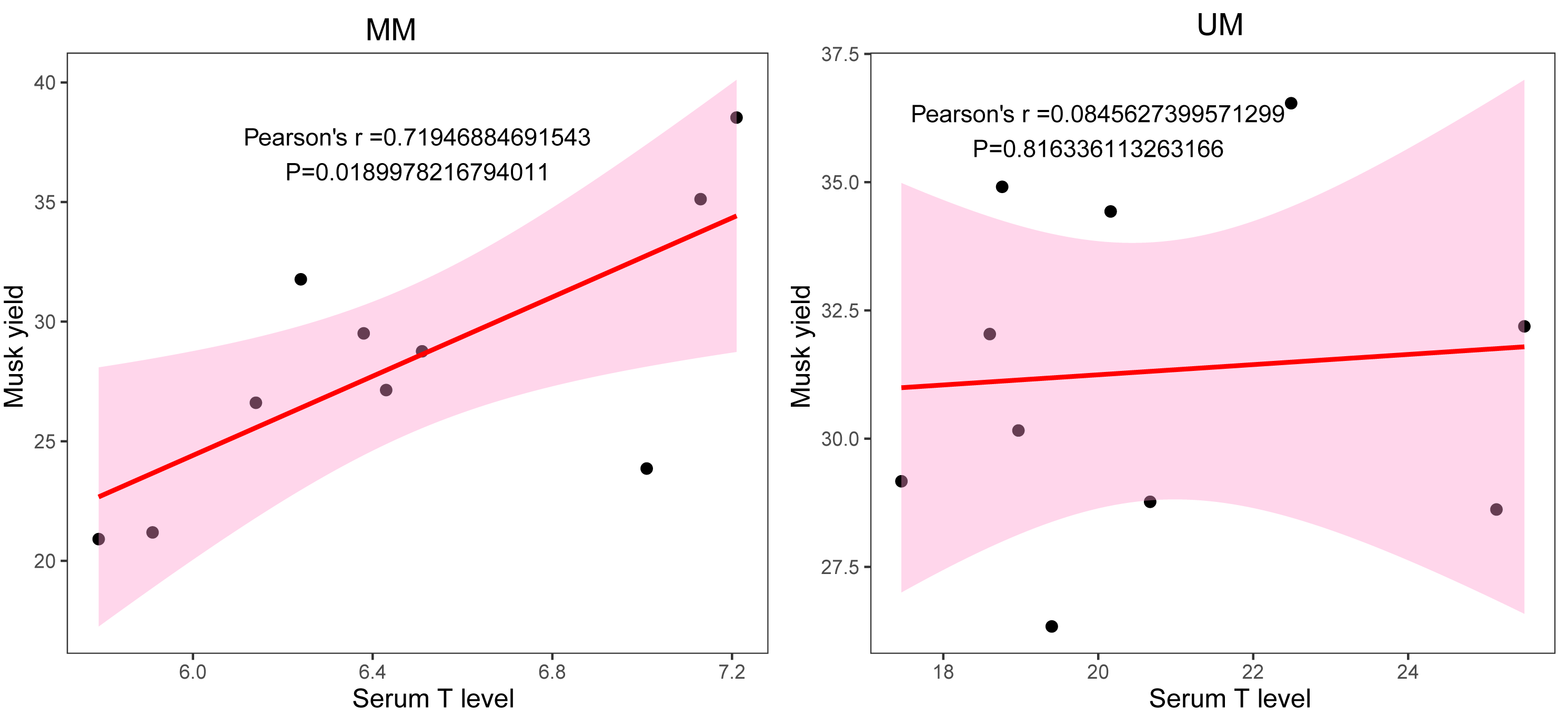

Supplement: Supplementary file 1 [file Image_1.TIF]

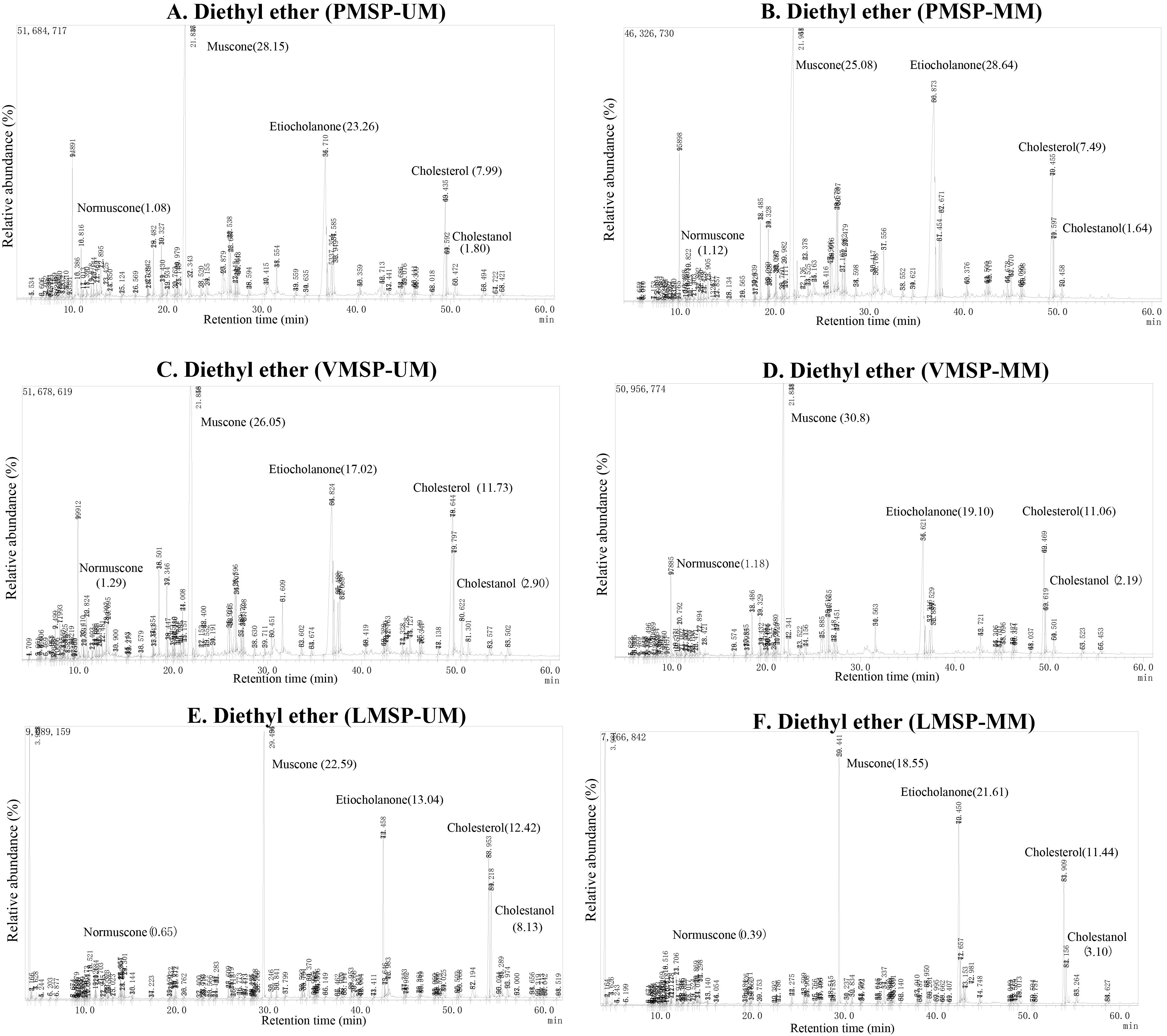

Supplement: Supplementary file 2 [file Image_2.TIF]

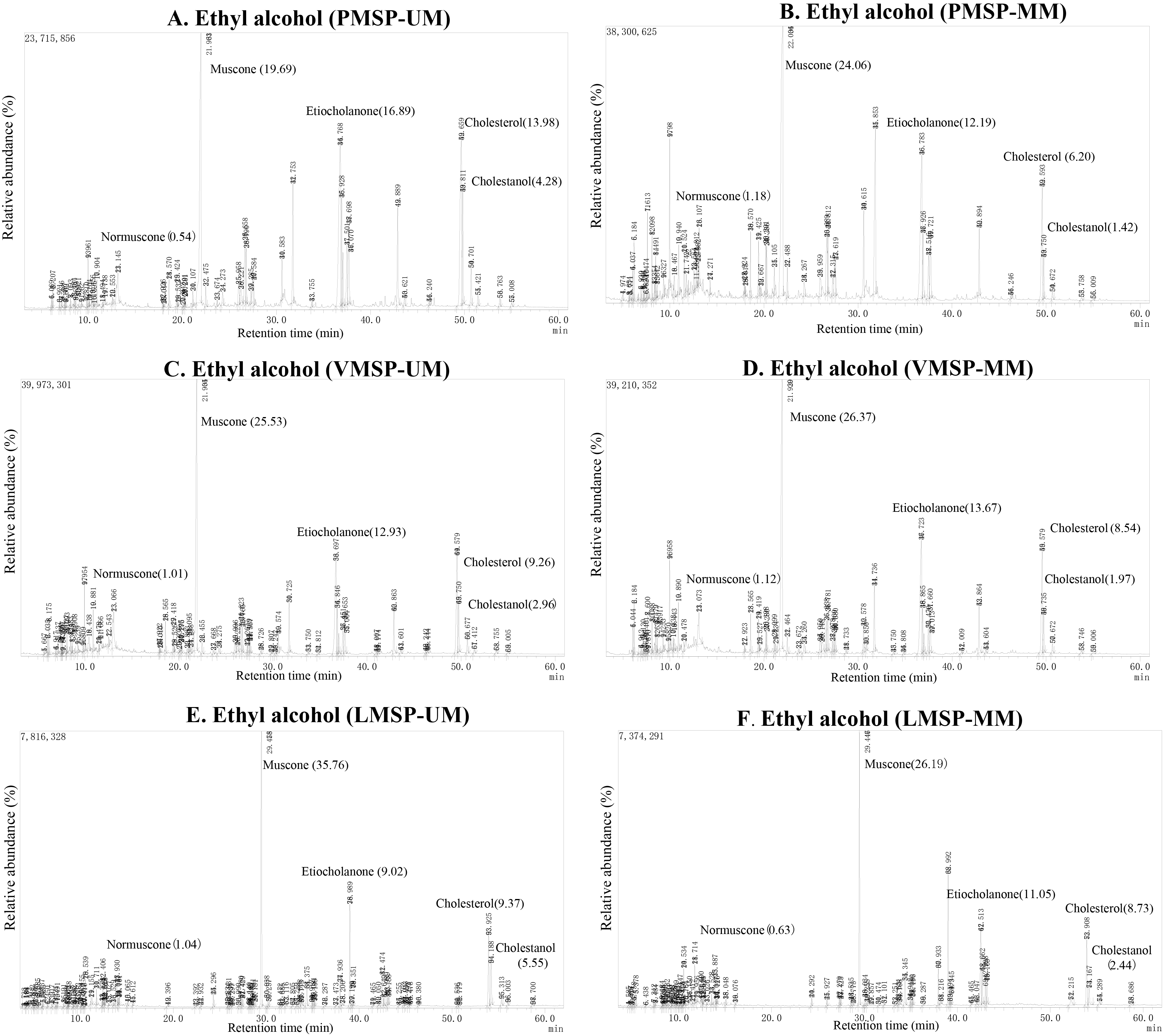

Supplement: Supplementary file 3 [file Image_3.TIF]

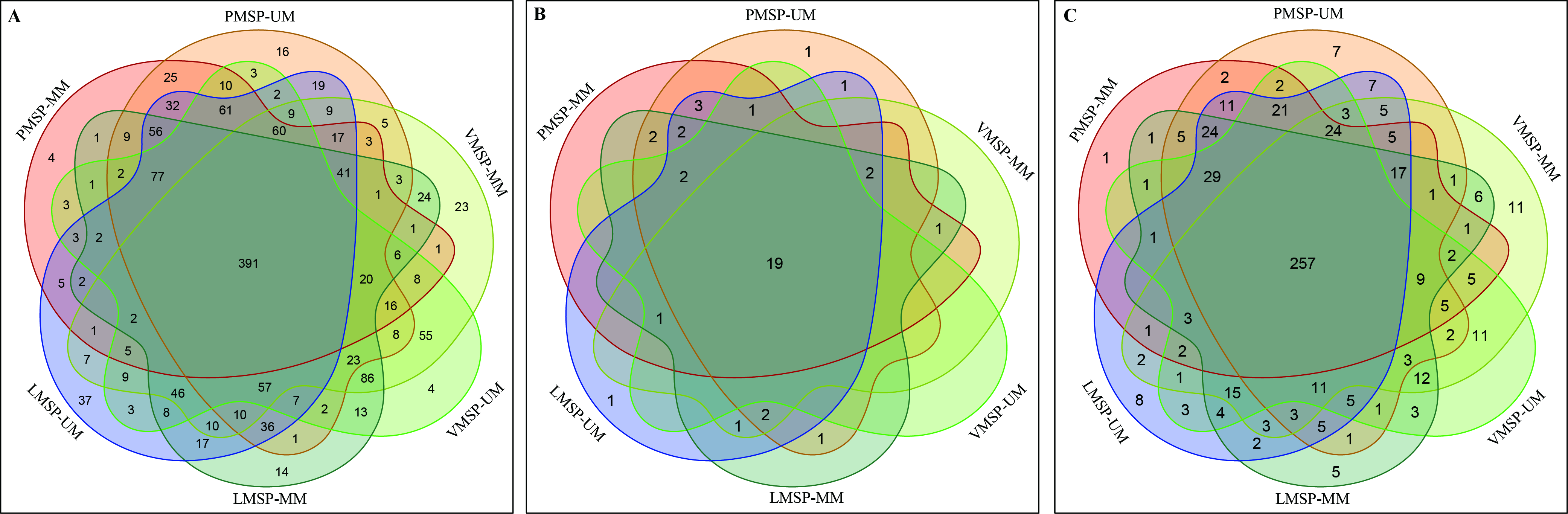

Supplement: Supplementary file 4 [file Image_4.TIF]
